# A Proteomics Outlook on the Molecular Effectors of CAR‑T Cell Therapy in Cancer Management

**DOI:** 10.1021/acs.jproteome.4c00930

**Published:** 2025-03-06

**Authors:** John Oluwafemi Teibo, Virginia Picanço-Castro, Lucas Eduardo Botelho de Souza, Vitor Marcel Faça

**Affiliations:** † Department of Biochemistry and Immunology, Ribeirão Preto Medical School, 28133 University of São Paulo, Ribeirão Preto, São Paulo 14049-900, Brazil; ‡ Center for Cell-based Therapy CTC, Regional Blood Center of Ribeirão Preto, University of São Paulo, Ribeirão Preto, São Paulo 14051-140, Brazil

**Keywords:** CAR-T Cell Therapy, Proteomics, Phosphoproteomics, Molecular Effectors, Mass Spectrometry/LC-MS/MS, Cancer Management

## Abstract

In the field of cell and immunotherapy, chimeric antigen receptor T-cell (CAR-T cell) therapy is the state-of-the-art therapy. It utilizes genetically engineered T cells expressing receptors against specific tumor cell targets such as CD-19 to induce cytotoxicity in and kill malignant cells. CAR-T cell therapy has demonstrated tremendous success in hematological cancers and promising results in solid tumors. However, CAR-T cell therapy has some limitations, such as causing cytokine release syndrome and neurologic abnormalities, leading to loss of target, and, most importantly, its high cost. Profiling the molecular mechanisms of CAR-T has provided important contributions to decomplexing biology and understanding more refined signaling and function of the cell therapy. Proteomics can elucidate alterations in effector molecules. We searched for CAR-T cell therapy-related molecular effectors in databases such as PUBMED and SCOPUS. We found based on our analysis, certain proteins (CD28, IFNG, IL-2, IL-5, CCL3, granzyme B, LCK, TNF-α, CD3E, CD80, B-Raf, ITK, and JAK2) that might be key effectors in the refinement of CAR-T cell therapy effectiveness. This review presents an overview of the key findings from proteomics studies related to CAR-T cell therapy, highlighting the critical proteins and signaling pathways involved in the therapy efficacy.

## Graphical abstract

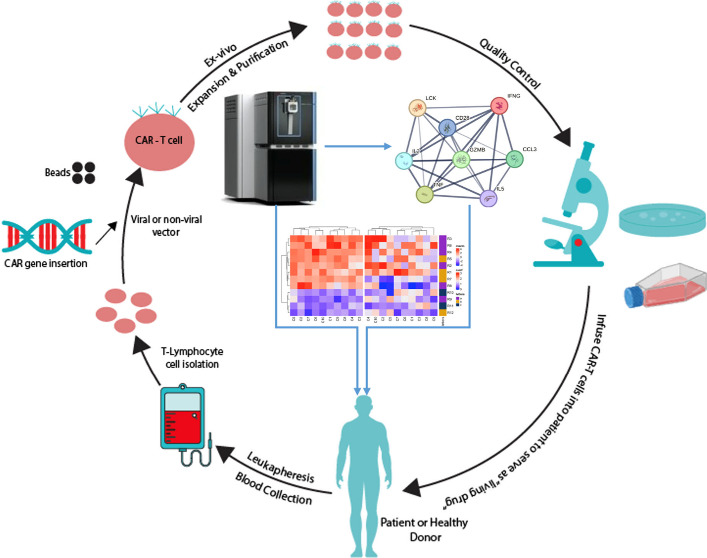

## Introduction

Chimeric antigen receptor T-cell (CAR-T cell) therapy has become one of the most promising personalized cellular therapies for a vast diversity of pathologies. CAR-T cell therapy utilizes genetically engineered T cells that express receptors against specific antigens present on tumor or unwanted cells, such as CD-19, BCMA, CD-23, CD-22, CD-33, or HER2, to recognize, induce cytotoxicity in, and kill malignant cells. Derived from either patient (autologous) or healthy donors (allogeneic) T cells, these modified cells serve as a living drug that can selectively destroy cancer cells.^1,2^

This therapy presents notable advantages, such as cell persistence, long-term survival in the host body, and cytotoxic activity. This therapy has achieved complete response rates, high clinical efficacy, and overall survival improvement in patients with hematological malignancies. Moreover, recent studies have attempted to adapt it for solid tumors, with the first engineered cell therapy for solid tumors being approved in August 2024.^3^ The first T-cell receptor (TCR) cell therapy was approved for use in people with specific human leukocyte antigen (HLA) types who had previously undergone chemotherapy for advanced MAGE-A4^+^ synovial sarcoma. For more than 10 years, the first novel therapeutic option for patients with synovial sarcoma was TECELRA.^3^ Although this therapy has various benefits, some drawbacks remain, including cytokine release syndrome, neurologic abnormalities, loss of target, anaphylaxis, poor trafficking and tumor infiltration, and lack of predictive biomarkers for response and toxicities.^4^ Additionally, high cost continues to remain a barrier limiting its widespread use.

Given the rise in clinical studies and the endorsement of multiple CAR-T cell therapy products, it is imperative to understand the underlying mechanisms of this treatment and the molecular effectors involved in the signaling pathways and the appropriate designs of CAR cell constructs to improve CAR-T cell therapy. In this context, the fundamental goal of molecular profiling is not only to identify the genes or proteins in a cell but also to generate a complete three-dimensional functional map of the engineered cell, including the activation, exact location, interaction, modification, and abundance^5^ of such molecules upon the activation of CAR-T cells in a target cell within a biological system.

This review presents an overview of the contribution of proteomics to the CAR-T cell therapy scene, showcasing major proteomics and phosphoproteomics studies that have been conducted to identify protein players and improve CAR-T cell design to advance cancer management. The various proteomics methods used to identify and map proteins, protein–protein interactions, molecular mechanisms of action, and signaling in nonsolid and solid tumors involved in CAR-T cell therapy are listed. In addition, key contributions from a proteomics perspective to the CAR-T development and application landscape are highlighted.

### CAR-T Cell Generation and Production

Before navigating into the proteomics changes produced in an engineered CAR-T cell, it is necessary to understand that key new functions are introduced in a cell using CAR constructs. These CARs comprise an antigen recognition domain (the humanized or murine single-chain variable fragment [scFv] of immunoglobulin), a hinge or spacer domain, one or more costimulatory domains (often CD28 or 4–1BB), the primary signaling domain (often the CD3z chain). In contrast to TCR-specific/high-affinity T cells, CARs use their scFv to bind to the target antigen without being HLA-restricted. First-generation CARs contain only the primary signaling domain, while second- and third-generation CARs incorporate additional costimulatory domains.^6,7^

Second- and third-generation CAR-T cells are more potent than “physiological” TCRs because the costimulatory domains of these CAR-T cells are directly attached to the CAR construct.^6,7^ Fourth-generation CAR-T cells have the armored “TRUCK” (T cells redirected for universal cytokine killing) with cytokines and costimulatory ligands that induce immunomodulatory changes, enhancing their action upon stimulation.^8^ The design and refinement of CARs are essential to balance the immune response; this is an area of great interest and fast development.

To produce a CAR-T cell and introduce the expression of the chimeric receptor in it, T-lymphocyte cells derived from blood samples obtained from healthy donors (allogeneic) or patients (autologous) are separated using apheresis a/leukapheresis and the CAR transgene is transfected using viral or nonviral vectors. Next, CAR-T cells are cultured and purified. Subsequently, quality control and quality assurance procedures are conducted to ensure that CAR-T cells meet the required safety, efficacy, and overall quality standards (Figure 1). These cells are then infused into the patients, acting as a “living drug” that targets specific antigens on the surface of malignant cells, destroying and killing them.^9^

**Figure 1:**
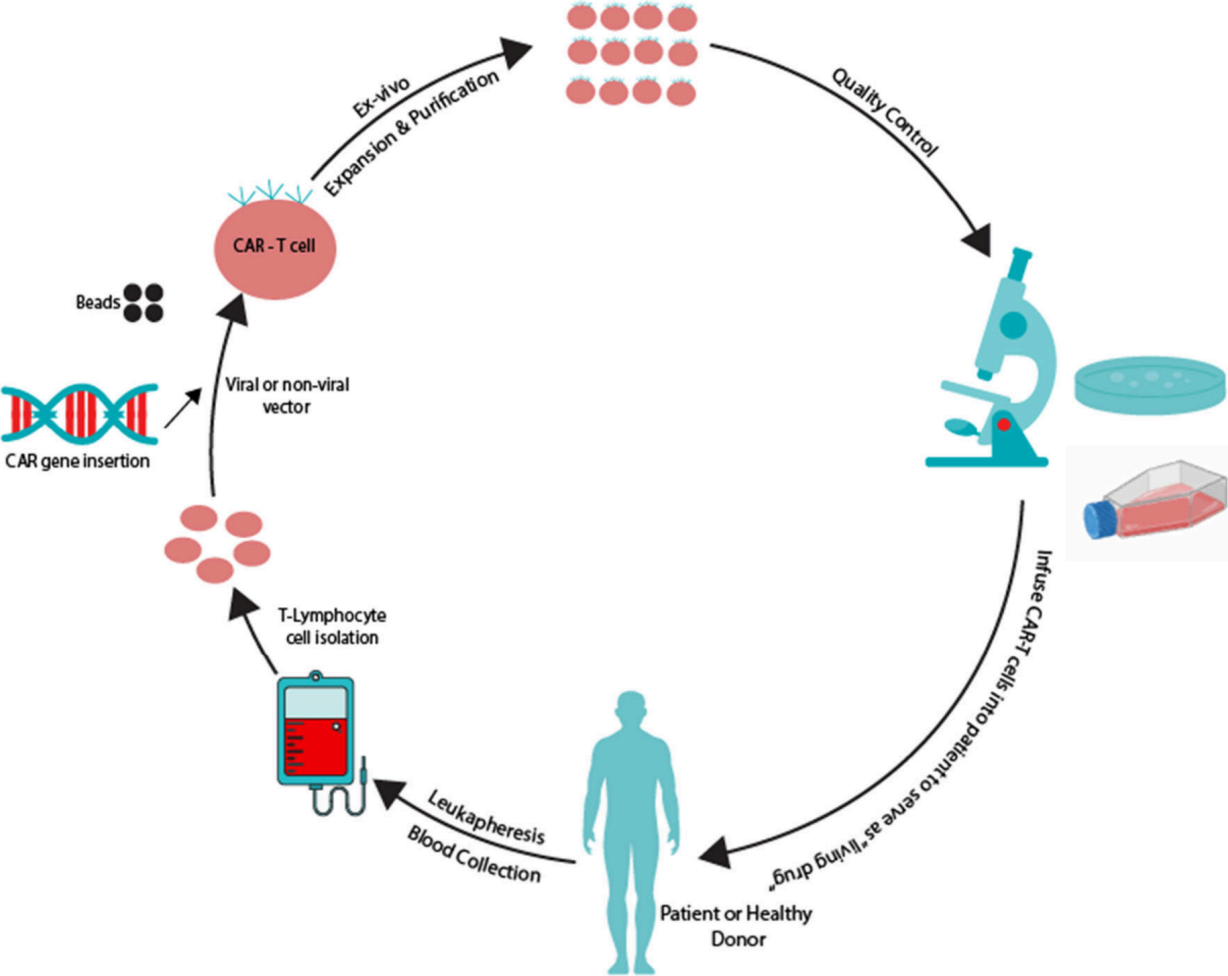
Illustrated process of CAR-T cell production.

## Proteomics

Proteomics give precise details about active proteins that have been post-translationally altered, and are crucial players in mediating numerous biochemical reactions, as well as cell expression and localization.^10,11^ Allowing accurate protein and peptide quantification, high-quality proteomics approaches have been developed to investigate biological changes at the protein level. Most proteome research studies on disease, signaling, or drug mechanisms used mass spectrometry-coupled high throughput liquid chromatography (LC-MS/MS). LC-MS/MS, yielding high-resolution experimental data, has allowed researchers to characterize previously indecipherable structural properties of proteins and protein assemblies.^11,12^

Currently, two analytical principles are employed: the targeted and global proteomics approaches.^13^ In global proteomics analysis, no specific proteins have been hypothesized to have changed. These methods are particularly interesting for clinical settings since they can quantify many proteins under various circumstances using either label-free or isotope-labeled methods.^13,14^ Targeted proteomics employs mass spectrometry to quantify a known and designated group of proteins. Throughout the experiment, panels made up of distinct peptide sequences that mirror the target protein are closely scrutinized. To improve ion sensitivity and precision, this technique depends on the high selectivity of peptide ions filtering.^10,15,16^

Continuous technological development has improved the depth, precision, and reproducibility of proteome measurements across samples. These techniques include real-time data analysis, FAIMS (Field Asymmetric Ion Mobility Spectrometry), and SPS-MS3 (Synchronous Precursor Selection-2nd Generation Product Ion Spectra). The label-free data-independent acquisition approach is starting to be applied in proteogenomic and multiomics studies, showing tremendous potential for decreasing the number of missing values in different samples and proteomics data.^17^

New technologies have led to the introduction of the Orbitrap Astral Mass Spectrometer, a “game changer” that shook up the proteomics field. It allows great mass accuracy, sensitivity, speed, and data resolution, allowing the analysis of 48 human proteomes or 100 yeast proteomes in a single day.^18,19^ The amazing front-line leading inventions are the Special time-of-flight timsTOF SCP and timsTOF Pro for trapped ions with an objective, and in-depth single-cell 4d-Proteomics. timsTOF SCP transformed quantitative single-cell biology research by permitting the analysis of hundreds of samples with a low sample load (<200 ng) at sequencing rates higher than 100 Hz and without sacrificing proteome depth.^20^

Based on these new developments and the continuous evolution of mass spectrometry technologies, data generated from this approach will help simplify biological questions and offer insight and answers that will ease the routine clinical applications of proteomics.

## The Contribution of Proteomics and Phosphoproteomics to CAR-T Cell Science

The proteomics view of molecular effectors involved in CAR-T therapy has just started to emerge. A recent study compared different CAR designs that targeted the same extracellular antigen on cancer cells but carried distinct intracellular signaling domains using certain proteomics-based techniques such as immunoproteomics and immunoprecipitation MS.^21^ It showed that it was the structure of the intracellular region that dictated the activity of CAR, not the choice of costimulatory molecules or the arrangement of the region where the receptor is attached to the cell. Furthermore, a thorough phosphoproteomics investigation revealed that certain CAR constructs could trigger T-cell signaling pathways downstream. These findings greatly influenced the practical application of synthetic immunology products and the development of next-generation CARs and combination therapies.^21^ Table 1 lists the proteomics and phosphoproteomics studies that contribute to CAR-T cell therapy.

**Table 1:** Overview of Studies That Used Proteomics and Phosphoproteomics to Understand and Improve CAR-T Cell Therapy

CAR Construct/Cells^ref^	Methodology	Main Proteomics Results	Proteins highlighted
Effector Discovery			
CD19-CAR T cells vs Raji B target cells^26^	Coculture CD19-CAR-Jurkat cells x Raji cells;	Coculture for 2 and 5 min captured much of the activation and deactivation of pTyr signaling;	LCK, ERK1/2, LAT, SLP76, CD 28, ZAP-70, Abl1/2, PLCy1
	Phosphoproteomics using SH2 domain and pTyr enrichment;	CD19-CAR activation promoted a global increase in tyrosine phosphorylation;	
	Quantitation SILAC-based.	Phosphoproteomics suggested divergence between early CAR and TCR signaling	
CD19-BB-28–3z CAR-T cells x Raji B target cells^27^	Coculture CD19-CAR-Jurkat cells x Raji cells;	Regulation of master transcription factor genes TBX21 and GATA3;	TBX21, GATA3, IFNG, TNFa, IL5, IL13
	Single-cell transcriptomics;	Regulation of signature cytokine representing mixed TH1/TH2 function in the same cell, rather than adhering to either TH1 or TH2 subtypes.	
	Single-cell proteomics.		
ROR1-specific 4–1BB/CD3ζ and CD28/CD3ζ CARs^25^	CAR T cells were cocultured with transduced K562 or MDA-MB-231 cells;	Phosphorylation of certain key downstream signaling intermediates is delayed and/or weaker after CAR stimulation than TCR stimulation;	CD3e, GRB2
	Global phosphoproteomics.	LCK was differentially associated with BB/ζ and 28/ζ CARs;	
		CD3ε and GRB2 sequences promoted the strongest and most durable antitumor functions.	
Bispecific LV20.19 CAR T-cell^23^	Stimulation with K562 cells CD19 or CD20	Effector cytokines were the main driver for differentiating cell subsets;	IFN-γ, granzyme B, MIP-1α, MIP-1β, perforin, IL-8, TNF-α and TNF-β
	Single-cell proteomics analysis.	CD4+or CD8+ CAR-T cells were not significantly different considering polyfunctionality scores;	
		Stimulation with either CD19 or CD20 antigens resulted in similar levels of analyte activation.	
LVαCD19–4–1BB-CD3ζ^28^	Single-cell proteomics (SCBC (IsoCode Chips)	IFN-γ, MIP-1α, IL-8 in both CD4 and CD8 T cells, granzyme B in CD8 T cells, and IL-17A and IL-5 in CD4 T cells were the main cytokines and chemokines generated by product polyfunctional T cells in the responding patients. These findings imply that the frequency and cytokine production levels of polyfunctional T cells in the product are linked to both the toxicity and clinical response to CAR T cell treatment.	IL-17A, IL-8, MIP-1α, IFN-γ, IL-5, granzyme B
Primary human CD8+ CD28/CD3ζ and 4–1BB/CD3ζ CAR T^24^	stimulation-induced phosphoproteomic changes	CAR constructs activated similar signaling intermediates;	IL-2, CD-28, LCK, GZMB, IFNG, TNF, CCL3, CCL4
		Stimulation of CD28/CD3ζ CARs activated an effector T cell-like phenotype and function;	
		4–1BB/CD3ζ CAR T cells preferentially expressed T cell memory–associated genes and exhibited sustained antitumor activity.	
PSCA- CD28/CD3ζ CAR-T cells^21^	CD8+ CAR-T cells infused in mice piercing HPAC pancreatic tumors;	Identification of 253 possible CAR interaction partners were identified;	TCR, CD28, PKCq, STAT3, IL-2, ENO1, TPI1, PKM, ACTG, FLNA, RRAS2, CD28
	Immunoproteomics;	15 canonical pathways associated with T-cell response enriched in this population.	
	Transcriptomics;		
	Immunoprecipitation (IP-MS).		
WT1 - CD28/CD3ζ CAR-T cell^29^	Treatment with 10 μM immunomodulatory drug Lenalidomide;	LEN enhances the effectiveness and antitumor effectiveness of WT1 CAR-T cells;	HLA-B, HLA-F, HLA-DQB1, HLA-DRB3, TRIP6, TRIP10, ANXA1, LGALS3BP, CD40LG, CD8A, CD6, ACTIN1, ACTN3, ACTR5, IL2RA, LGALS3BP, ACTIN1, TMEM65, FDXR, NDUFS5, MTCH1
	iTRAQ quantitative mass spectrometry analysis;	Strong upregulation of proteins involved in T-cell activation;	
		combination therapy with LEN and WT1 CAR-T may synergistically evoke a potent and durable antitumor response.	
Target Discovery			
B-ALL cell lines (MLLr cell line)^30^	N-glycoproteins enrichment of the cell surface proteins;	Cell surface proteomics revealed CD72 as an optimal target for poor-prognosis KMT2A/MLL1-rearranged (MLLr) B-ALL;	CD-72
		Modulation of known hallmarks of MLLr including PROM1 and FLT3 upregulation as well as loss of CD10;	
		Upregulation of several adhesion molecules, such as NCAM1, L1CAM, and ITGAV	
LVαCD19–4–1BB-CD3ζ^31^	Single-cell transcriptomics;	Substantial heterogeneity in the antigen-specific activation state;	IFNG, IL2, IL4, IL5, IL9, IL13, CCL3, CCL4, CCL5, IL16, IL32
	Cell Surface subcellular proteomics;	Deficiency of T helper 2 function was associated with CD19-positive relapses;	
	Multiplexed secretomics assay.	Functional immune programs in activated in CAR-T cells do not distinguish patients with CD19-positive relapse from complete responders.	
AML cell line, Human T Cells samples^32^	Cell surface-specific proteomics in a diverse panel of AML cell lines;	Identification of potential targets expressed in leukemia stem cells, but not in normal CD34+CD38- hematopoietic cells;	LILRB2, ADGRE2, CCR1, CD96, CD70, TNFRSF1B, CD96, CD123, CLEC12A and CD33
	Computational transcriptomics and proteomics data integration.		
Triple-negative breast cancer (TNBC) that are HLA-A2-positive^33^	Immunopeptidomics;	Immunopeptidomic identification of 19 675 peptides from tumor and adjacent normal tissue;	PCNA, IL-32, RPN2I, SDCBP, CFL1
	HLA-peptides profiling	HLA-A*0201- binding peptide sequences were shared between all patients;	
		Protein antigens were identified as having high cohort presentation coverage.	
His10-KKCK-CD3z, HIS10-LCK-G2A, HIS10-CD28-CD3z CARS transduced to HE^34^	Computational analysis;	The six tyrosine sites on CD3ζ are phosphorylated by LCK with different kinetics;	CD28, LCK
	Phosphoproteomics mass spectrometry	The total rate of CD3z phosphorylation is increased when CD28 is added to the N-terminal of CD3z;	
		LCK phosphorylates CD3z via a competitive inhibition mechanism.	
CD4+ or CD8+ CAR-T cells^35^	High multiplexing (single-cell 32-plex proteomics).	The CD19 antigen increased the levels of several cytokines;	Granzyme B, IL-2, CD137, IL-17A, IL-5, IL-8, IL-9, MIP-1β, IL-10, IL-4, Perforin, TNF-α, GM-CSF
		Granzyme B, IFN-γ, MIP-1α, Perforin, and TNF-α are effectors cytokines;	
		GM-CSF, IL-2, IL-5, IL-8, and IL-9 are stimulatory cytokines;	
		MIP-1β is chemoattractive;	
		sCD137, IL-10, and IL-4 are regulatory;	
		IL-17A is inflammatory.	

A very recent review highlighted the roles of single-cell omics technologies, namely single-cell genomics, transcriptomics, proteomics, and epigenomics, in relation to CAR-T cell therapy; it pointed out how single-cell analysis may be an effective tool for unlocking the mysteries of the tumor microenvironment in order to treat cancer with immunotherapy. The ability of that study to identify the best location for CAR transgene integration is impressive, as it may impact the persistence and effector function of CAR-T cells when CAR integration occurs at specific locations. Using CAR-T cell single-cell RNA sequencing (scRNA-seq) data, genes associated with a favorable clinical response can be identified and mapped.^22^ Some of the weaknesses of single-cell omics in CAR-T cells include variations in scRNA-seq that require the collection of hundreds or thousands of events to represent a particular sample, as well as the occasionally hazy link between gene expression and protein expression.^22^

A major frontier technology is single-cell proteomics using nano LC-MS, which has the potential to overcome some of the challenges of scRNA-seq and can be applied to CAR-T cell products, tumor microenvironment (TME), or any other tissue involved in the treatment process. Single-cell data can be used for various purposes, including creating atlases of tumor or CAR-T cells or their molecular changes, and correlating them with treatment outcomes. Single-cell analysis can also serve to identify the mechanisms of CAR-T cell therapy resistance in immune cells or TME, identify the mechanisms of CAR-T cell toxicity, demonstrate the impact of CAR gene integration on T-cell effectiveness, reveal and contrast TME features prior to and during CAR-T treatment, and establish links between treatment resistance, recurrence, or clinical outcome and the omics profile of CAR-T or tumor cells. It can also help identify the parameters associated with CAR-T or TME that lead to CAR-T cell exhaustion, assess the quality of CAR-T products *in vitro*, perform preliminary screening to identify patients who would benefit the most from CAR-T therapy, identify new cell populations and molecular protein effectors in TME or CAR-T cell products, and assess potential harm from CAR-T cell therapy to organs.^22^

Zurko et al.^23^ used single-cell proteomics analysis to conduct a thorough functional evaluation of polyfunctional CAR-T cells in a dual-targeted LV20.19 CAR-T-cell product. They found, that similar to CD19-activated CAR-T cells, CD20-activated LV20.19 had a 866–1109 polyfunctionality strength index (PSI), indicating that these cells retain their cytotoxicity against CD-19-negative tumors. This study also revealed that increased TGF-β and interleukin (IL) 17A (cytokines produced by CD4^+^ CAR-T cells) levels were strongly linked with clinical response. Single-cell proteomics and multiomics studies indicated that the presence of polyfunctional CAR-T cells that coproduce Th1 and, more crucially, Th2 cytokines may collectively contribute to a partial or complete remission in patients with B-cell malignancies. Nevertheless, various intrinsic and extrinsic characteristics of CAR-T cells can determine the clinical effectiveness and result of CAR-T cell therapy. For example, dual-specific CAR-T cells exhibit higher levels of polyfunctionality and cytokine production than monospecific CAR-T cells.

Understanding the molecular mechanisms and signaling pathways involved in the cytotoxic activity of CAR-T cells is crucial to design effective and persistent CAR constructs and to overcome bench-to-bedside challenges. Salter et al.,^24^ gave insight into phosphorylation events using peripheral blood mononuclear cells and LC-MS/MS. Like signaling intermediates, both CAR constructs (4–1BB/CD3ζ CAR-T and CD8^+^ CD28/CD3ζ CAR-T) were activated. A phenotype and function similar to those of effector T cells were connected with faster and larger-magnitude alterations in protein phosphorylation, which was activated by CD28/CD3ζ CAR stimulation. Meanwhile, 4–1BB/CD3ζ CAR-T cells demonstrated persistent anticancer efficacy against well-established tumors *in vivo* and selectively expressed genes linked with T-cell memory. The authors found out that signal strength is a major factor in the fate of T cells and that the CAR signaling pathways cannot be exclusively predicted by the domains making up the receptor. Therefore, they reduced toxicity and improved clinical performance by tailoring CAR design based on signal strength. Some of the main protein effectors identified in macrophages were IL-2, CD-28, lymphocyte-specific protein-tyrosine kinase (LCK), tumor necrosis factor-α (TNF-α), granzyme B, interferon-γ (IFNG), inflammatory protein 1α (CCL3), and inflammatory protein 1β (CCL4).

The same group used global phosphoprotein signaling to study bispecific T cells. They discovered that variations in the phosphorylation of linker for activation of T cells (LAT) and the conventional T cell signaling components, CD3δ, ε, and γ chains, were revealed by TCR and CAR signaling in identical T cells. When stimulated by CARs rather than TCRs, these proteins were either not phosphorylated at all or phosphorylated less strongly. The signaling data showed that additional research was needed to clarify the exact processes of CAR-T cell activation and implied that CARs may compensate for poor LAT phosphorylation by supraphysiologically phosphorylating CD3ζ, CD28, or other proteins. The authors determined that novel signaling and protein-binding domains can be logically inserted to increase CAR signaling and effector function. Compared with the inclusion or selection of alternative costimulatory domains, which have up to now been the primary focus of optimization in the CAR study, the insertion of such domains may enhance receptor signaling.^25^

Recently, Griffith et al.^26^ cocultured SILAC-labeled CD19-expressing Raji B cells and CD19-CAR Jurkat T cells (CD19-CAR-T cells), integrating the SILAC with minimal overlapping sites. Among the enrichment approaches, the sSH2 enrichment method altered pTyr sites (251 total, 238 distinct) more than the Ti0_2_ method altered pSer or pThr (44 total, 31 unique). The authors concluded that CAR activation by Raji B cells enhanced signal transduction in CD19-CAR-T cells via classical TCR and CAR-specific pathways. In reaction to CAR interaction, they observed a reduction in global phosphorylation in Raji B cells, which may be crucial to the therapeutic effectiveness of CD19-targeting CARs. The main protein players identified included LCK, ERK1/2, LAT, SLP76, CD 28, ZAP-70, Abl1/2, and PLCy1. Table 1 gives more details on phosphoproteomics studies.

## Methodology

We searched relevant databases, such as PUBMED, Google Scholar, and SCOPUS, using the keywords “CAR-T cell therapy” and “Proteomics” and “CAR-T cell therapy” and “Phosphoproteomics”. We found 106 articles on CAR-T cell and proteomics, and 5 articles on CAR-T cell and phosphoproteomics, searched between November, 2023 and April, 2024. We selected articles that identified specific proteins as molecular players or effectors of the cell therapy. We eliminated review articles and worked only with research articles. We also eliminated articles that utilized proteomics to study toxicity associated with CAR-T cells. We finally selected 14 articles on the molecular effectors, mechanisms, and signaling pathways of the CAR-T cell therapy. From these articles, we produced tables of the molecular effectors of CAR-T cell therapy. Among the 14 selected studies, 10 applied proteomics to map molecular effectors and 4 applied phosphoproteomics to map molecular effectors of CAR-T cell therapy.

## Proteomics Results

There is a compelling need to map the protein effectors, signaling pathways, and possible action mechanisms of the various constructs of the CAR-T-based strategies, as well as their variability and therapeutic efficacy. Considering published articles that conducted proteomics analysis on CAR-T cells, we sought to identify and characterize the recurrent and potentially relevant proteins involved in the cell therapy mechanism. Our search yielded 14 original research articles that focused on the molecular effectors of the CAR-T cell therapy, as described in Table 1.

### Selected Studies Used Various CAR-T Cell Constructs

The selected studies used various types of CAR-T cells of the first, second, and third generations (Figure 2). Most studies used one type of CAR-T cell, while some, like Salter et al.^25^ and Ramello et al.^21^ used multiple types of CAR-T cells. The CAR-T cell constructs belong to three major categories based on antigen recognition domains (CD19, FMC63, and PSCA) and other minor categories (for example, constructs with CD72 as the antigen recognition domain). Irrespective of the CAR-T cell constructs, the molecular effectors can also differ. Thus, we documented the dominant and common molecular effectors reported in these studies.

**Figure 2:**
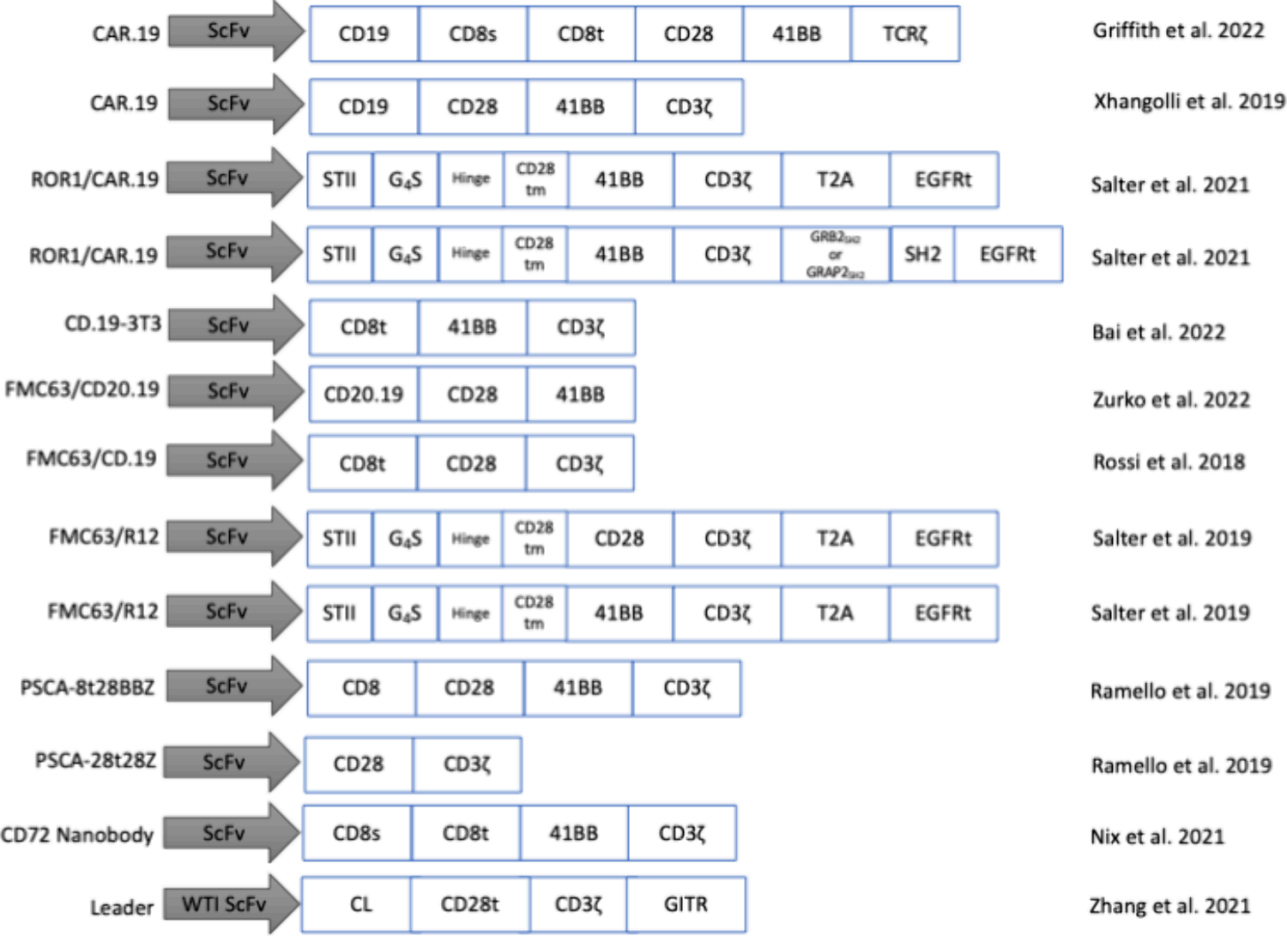
Overview of the various CAR-T cell constructs used in the selected studies.^36,37,42,43,44,46,47^

Table 1 compiles the main results of the articles that used proteomics and phosphoproteomics to explore various aspects of the CAR-T cell therapy used in the treatment of various cancers with the same aim to identify critical proteins and understand the signaling and action mechanism of this cell therapy. The first eight studies are CAR-T targeted studies that explore various molecular effectors using proteomics approaches, while the last six are CAR-T functional studies that explore target discovery using computational biology, single-cell proteomics, and other functional approaches.

### Overview of Important Targets Identified by Proteomics Studies

As highlighted in Table 1, proteomics and phosphoproteomics studies identified several proteins involved in T-cell effector function and activation as regulated during CAR-T response. As an example, in a bid to map interesting protein targets across the various studies, we examined the main proteins modulated from all the articles and observed the top eight proteins identified and validated from at least three articles. The top recurrent proteins observed were CD28, IFNG, IL-2, IL-5, CCL3 (Chemokine (C–C motif) ligand 3 (CCL3)/macrophage inflammatory protein 1-alpha (MIP-1-alpha)), granzyme B, LCK and TNF-α, all associated with T-cell activation as expected. This indicates that proteomics can be used to discriminate the process of T-cell activation with precision based on the regulated molecular effectors.

Figure 3 and Table S1 show the interaction network of these top recurrent proteins, their close correlation from at least three studies, and the biological process and molecular function involved, based on our own analysis and using the STRING database. An overview of the molecular effectors, their biological role, and signaling pathways involved in the activity of CAR-T cells is described below.

**Figure 3:**
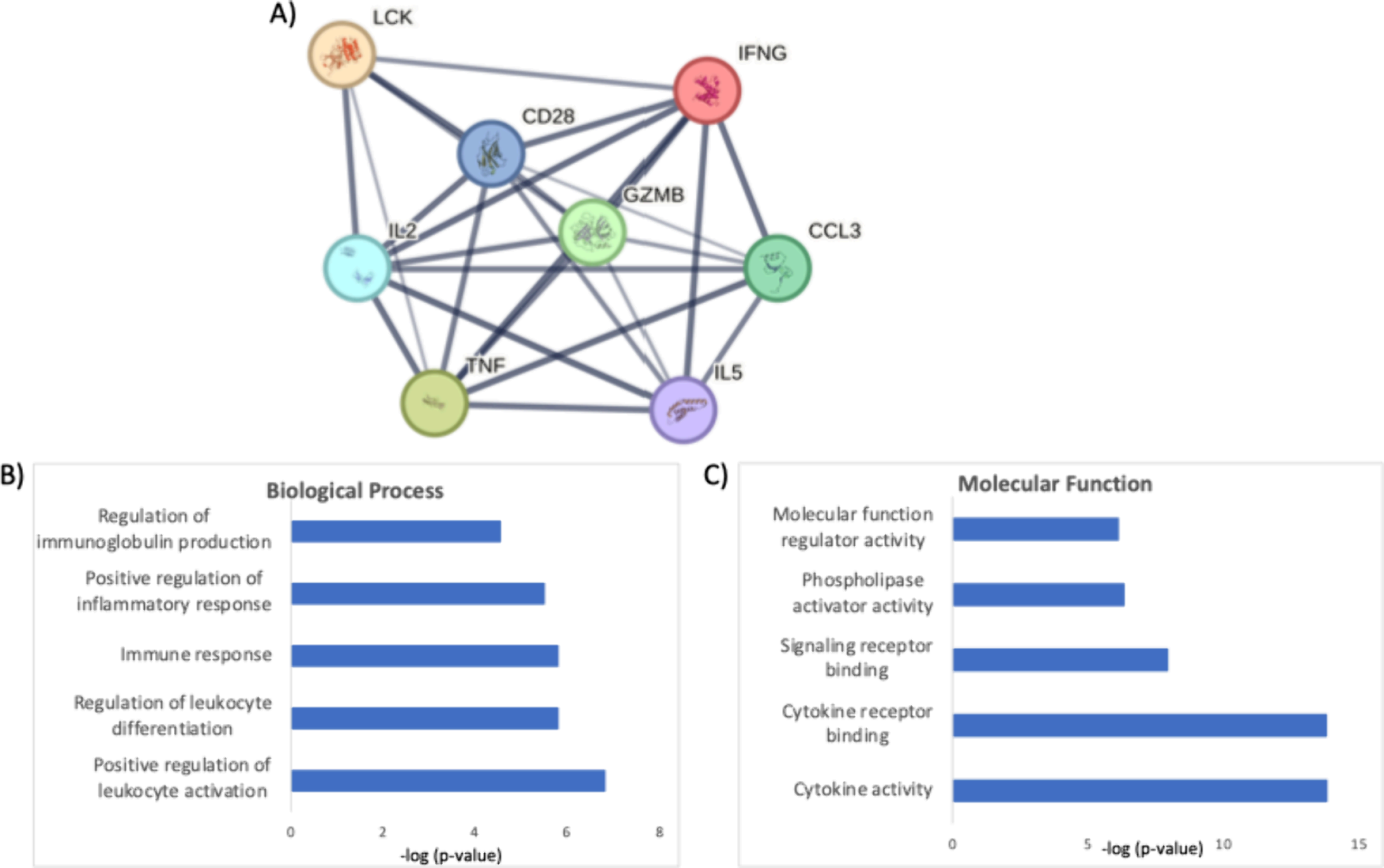
(A). Interaction network of the top recurrent proteins modulated, based on the selected studies. (B) and (C) show the profiles of the top biological processes and molecular functions, respectively.

These proteins and their role as molecular effectors in the activation and cytotoxicity of CAR-T cell therapy are discussed in detail below.

### Overview of the Molecular Effectors, Their Biological Role, and Signaling Pathways Involved in the Activity of CAR-T Cells

The identified (Molecular effectors CD28, IFNG, IL-2, IL-5, CCL3/MIP-1-alpha, Granzyme B, LCK and TNF-α) are either cytokines, receptors, kinases, proteases, or chemical messengers.

### Cytokines

#### IFNG

IFNG, also called type II interferon, is produced by immune cells and it stimulates effector immune cells and promotes antigen presentation. It primarily affects gene expression through the JAK-STAT signaling pathway upon contact with its receptor IFNGR1. Upon IFNG binding, the intracellular domain of IFNGR1 opens up, enabling its interaction with downstream signaling components. It enhances the anticancer activity of the CAR-T cell therapy (36–37).

#### IL-2

IL-2 drives critical functions in the immune response and tolerance; it is produced by activated CD4-positive helper T cells, activated CD8-positive T cells, and natural killer cells to a lesser extent. It binds to IL-2R or an IL2RA/CD25 receptor complex. Following interaction with the receptor, the IL-2R subunits oligomerize and initiate downstream signaling through JAK1 and JAK3 phosphorylation. Then, JAK1 and JAK3 phosphorylate the receptor, creating a docking site that phosphorylates many substrates, including STAT5. IL-2 stimulates CAR-T proliferation and effector activity.^38–41^

#### IL-5

IL-5 is a cytokine produced by natural killer cells and T lymphocytes and is crucial to eosinophil survival, differentiation, and chemotaxis. It stimulates the development, differentiation, and synthesis of immunoglobulins in resting and active B cells. Its biological effects are mediated via a receptor that comprises the cytokine receptor common subunit beta (CSF2RB) and the IL5RA subunit. Upon binding, its receptor activates several kinases, such as LYN, SYK, and Janus Kinase 2 (JAK2). This signal propagates through the RAS-MAPK and JAK-STAT5 pathways. This enhances CAR-T cell response chemotaxis and immunoglobulin production and activation (42–44).

#### CCL3

CCL3 is a monokine with chemokinetic and inflammatory characteristics that binds to the CCR4 and CCR5 receptors; it is one of the main substances produced by CD8+ T cells that suppress viruses that drive mutation in cancer. As a member of the CC chemokine family of cytokines, CCL3 contributes to the acute inflammatory state by binding to the receptors CCR1, CCR4, and CCR5 and attracting and stimulating polymorphonuclear leukocytes. CAR-T cells drive this inflammatory process.^45^

### Receptors

#### CD28

CD28 is a receptor promoting T-cell activation, proliferation, and survival. Its mechanism of activation involves the recruitment of GRB2 and protein kinase C-theta/PRKCQ, resulting in the activation of NF-κ-B through both PI3K/Akt-dependent and -independent pathways. This enhances the cytotoxicity and survival of CAR-T cells (46–47).

### Kinases

#### LCK

When TCR binds to peptide antigen–bound MHC complexes, the matching LCK protein is transferred to the area around the TCR/CD3 complex. LCK phosphorylates immune tyrosine-based activation motifs, which are found in the cytoplasmic tails of TCR-gamma chains and CD3 subunits. Upon stimulation, TCR recruits the tyrosine kinase ZAP70, which LCK phosphorylates and activates, and subsequently, ZAP70 activates multiple signaling molecules, generating lymphokines. These lymphokines immediately bind to the CD2 cytoplasmic tail, causing hyperphosphorylation and LCK activation. This phenomenon contributes to the signaling cascade related to the IL-2 receptor, which regulates the T-cell proliferative response. In addition, the LCK signaling pathway leads to the gene expression of transcription factors such as NFKB, Jun, and FOS, which mediate the continuous antitumor activity of CAR-T cells.^48,49^

### Proteases and Chemical Messengers

#### Granzyme B

Granzyme B is a protease that enters target cells through the immunological synapse and activates caspase; it is found in the cytosolic granules of cytotoxic T and natural killer cell–independent pyroptosis. Following Asp phosphorylation, granzyme B accelerates the cleavage of gasdermin-E once it has entered the target cell. This results in pyroptosis and releases the pore-forming moiety of gasdermin-E. The caspase activation cascade—a set of aspartate-specific cysteine proteases that trigger the execution of apoptosis—seems involved in target cell death. Granzyme B cascade yields active enzymes that mediate apoptosis by cleaving caspase-3, −9, and −10. In reaction to bacterial infection and foreign antigens, granzyme B cleaves and activates caspase-7, facilitating the repair of the plasma membrane. CAR-T cells utilize granzyme B to kill malignant cells in tumors.^50–52^

#### TNF-α

TNF-α is a TNFRSF1A/TNFR1 and TNFRSF1B/TNFBR-binding cytokine. It can kill certain tumor cell types and is mostly secreted by macrophages. It is a strong pyrogen that can directly cause fever or stimulate the release of IL-1, and can also cause cachexia. In some situations, it can also promote cell differentiation and proliferation, reducing the function of regulatory T (Treg) cells in patients with rheumatoid arthritis by dephosphorylating FOXP3. The phosphorylation of Ser418 on the *C*-terminal DNA-binding domain controls the suppressive activity of Treg cells. TNF-α can also elevate the expression and enzymatic activity of protein phosphatase 1 in the inflamed synovium, promoting the dephosphorylation of the Ser418 site in Treg cells generated from rheumatoid arthritis, impairing Treg cell function. Additionally, TNF-α is an important mediator of BCG-stimulated neutrophil-mediated tumor cell death in combination with a DIABLO/SMAC mimetic in the RT4v6 bladder cancer cell line. It mediates cell death and IL-1 release, mediating the cytotoxic activity of CAR-T cells. It also mediates CAR-T cell toxicity, endothelial activation, and proinflammatory responses.^53–56^

All these proteins play major roles in the effector function of CAR-T cell-induced cytotoxicity and tumor cell death and for the persistence of CAR-T cells *in vivo*.

## Phosphoproteomics and Signaling

In an attempt to map interesting protein targets across the selected articles, we examined the phosphoproteomics data set generated by.^21,24–26^ From these four articles, we generated a list of molecular effectors identified through phosphoproteomics and studied the involved signaling pathways. We found about 438 modulated phosphoproteins that appeared at least twice out of the four data sets and mapped their associated pathways and biological functions. Based on the STRING database, the pathways included TCR activation, activation and regulation of the immune system, apoptosis signaling, and regulation of cytokine production (Figures 4 and 5, Table S2). This result aligns with what was expected and demonstrates that the phosphoproteomics strategy efficiently identifies the effectors of the CAR-T activation process.

**Figure 4:**
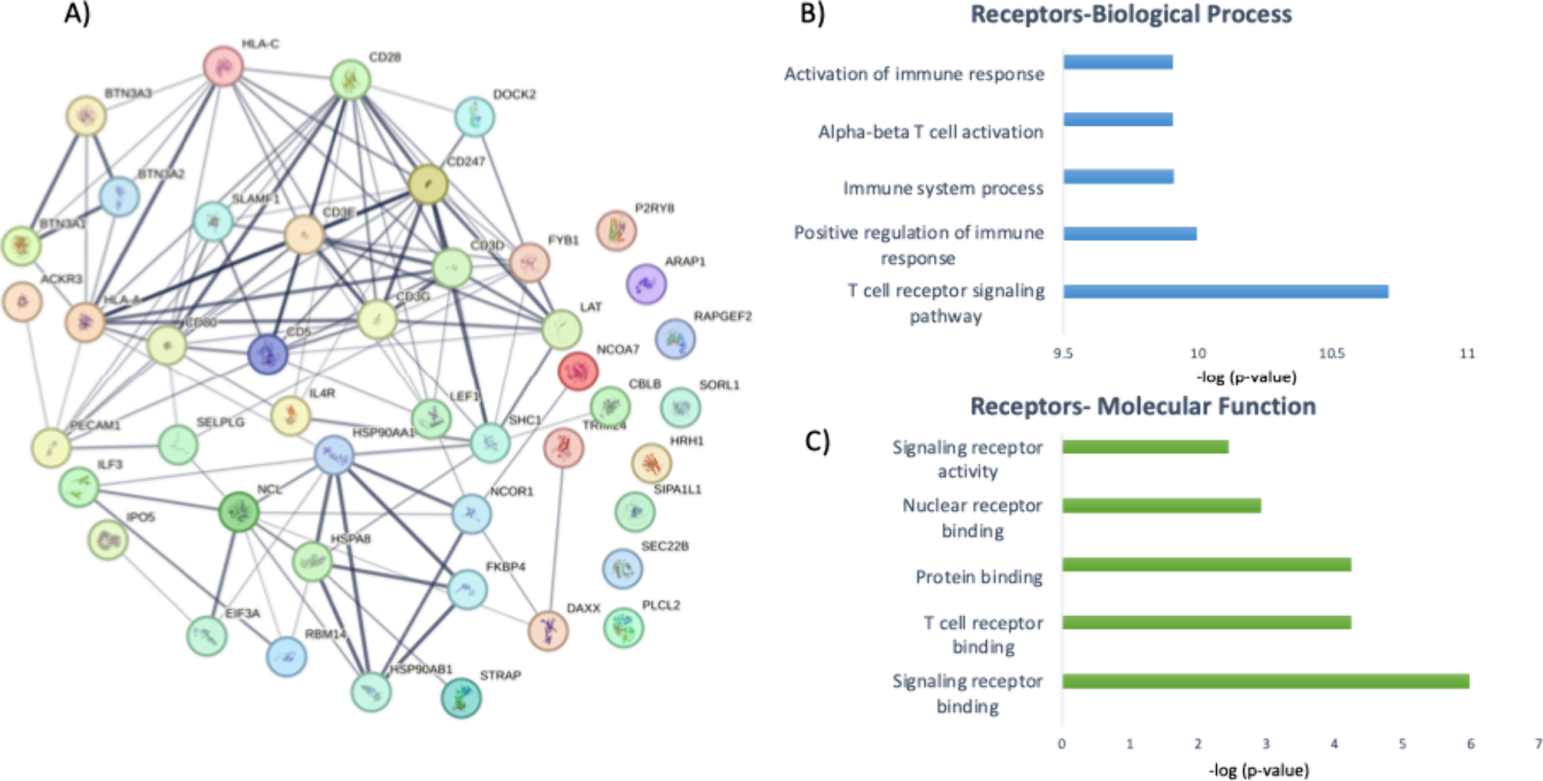
(A). Map of interconnected receptor proteins from the phosphoproteomics study listed above. (B) and (C). Biological process and molecular function profiles of the receptor proteins.

**Figure 5:**
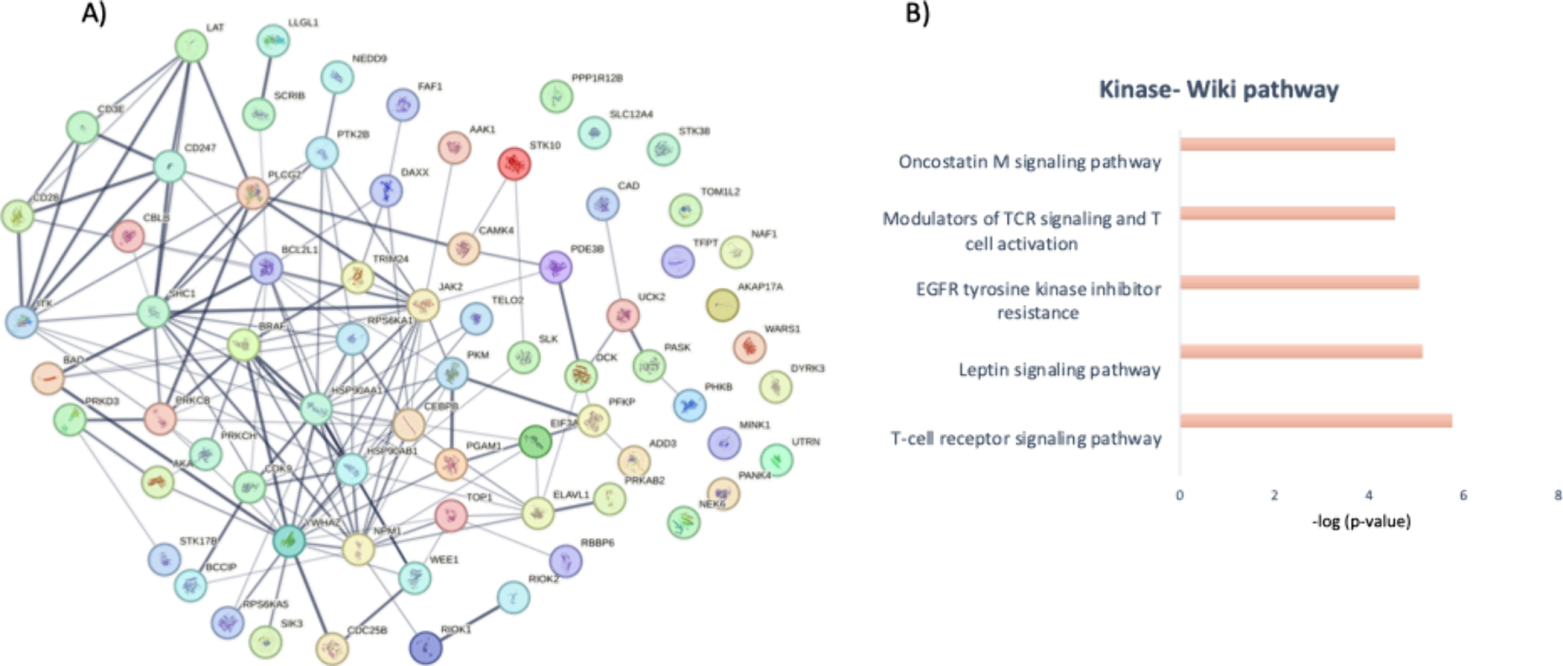
(A). Map of interconnected kinase proteins from the selected phosphoproteomics studies. (B). Wiki-pathway profile of the kinases.

In these lists of proteins, we explored the receptors and kinases among the phosphoproteins and identified 45 proteins that are receptors and 71 kinases that are molecular effectors of the CAR-T cell therapy (Table S3). Note-worthily, some of these proteins, such as CD28, C247, CD3E, LAT, SHC1, and DAXX, are both receptors and kinases. Some of them, like CD3E, CD247, and CD28, are strongly associated with T cells, while others, such as SHC1 or DAXX, are not directly associated with T-cell function, but our analysis mapped them as phosphoproteins that mediate CAR-T cell function. Finally, we identified kinases such as B-Raf, IL-2-inducible T-cell kinase (ITK), and JAK2.

Many T-cell membrane proteins and nonreceptors highlighted above play important roles in signal transduction for CAR-T cell signaling. Some of the identified receptors, such as CD28, CD3E, and CD80, are discussed below.

CD28 is a receptor that aids in IL-2 production and is critical to T-cell survival, proliferation, activation, and effector activity. These effects enhance the cytotoxicity and survival of CAR-T cells. CD3E is a component of the TCR-CD3 complex, which is found on the surface of T cells and is crucial to the adaptive immune response. TCR-mediated signals propagate across the cell membrane through the CD3 chains CD3D, CD3E, CD3G, and CD3Z when antigen-presenting cells activate the TCR. Immune receptor tyrosine-based activation motifs are present in the cytoplasmic domain of all CD3 chains.^57^

CD80, a costimulatory molecule that is a member of the immunoglobulin superfamily, is crucial to the activation of T lymphocytes^58^ and acts as the main auxiliary signal that enhances the MHC/TCR signal in T cells constitutively expressing the CD28 receptor on their surfaces.^59^ Patients taking tisagenlecleucel for relapsed/refractory diffuse large B-cell lymphoma have increased levels of CD80 and/or CD86 in their tumor tissue. This finding helped the development of the CAR/CCR (chimeric checkpoint receptor) architecture, which consists of a recombinant CTLA-4-linked receptor with a 4–1BB costimulatory domain coexpressed with a first-generation CD19-specific CAR. *In vitro* investigations employing nonmalignant CD19+ cells demonstrated that CAR/CCR T cells were more selective, had better long-term activity, and were more effective in xenograft mouse models than CAR-T cells. Furthermore, following CAR/CCR T-cell therapy, immunocompetent animals exhibit an intact CD80–CD19+ B-cell population. Based on these results, the CAR/CCR design provides a viable approach for additional translational research.^60^

Next, we discuss the kinases B-Raf, ITK, and JAK2, which are effector kinases of the CAR-T cell therapy.

B-Raf is a serine/threonine protein kinase that plays a role in the transmission of mitogenic signals from the cell membrane to the nucleus. The MAP kinase signal transduction pathway is activated when MAP2K1 is phosphorylated.^61^ As individual agents, all B-Raf and MEK inhibitors decreased this cytokine release. Specifically, Dabra was the least potent inhibitor, and Dabra + Tram was significantly less potent than Vem + Cobi. The increase in the levels of CD25 and CD69 activation markers in CAR-transfected T cells following stimulation with antigens exhibited a similar pattern. Vem + Cobi drastically reduced CAR-T cell proliferation; however, the other kinase inhibitors exhibited little effect. Thus, the Dabra + Tram combination was preferable over Vem + Cobi to use with T-cell-based immunotherapy.^62^ Dabra + Tram might contribute to the decrease in cytokine release syndrome linked to CAR-T cell activity.

ITK is a tyrosine kinase essential for regulating the adaptive response of the immune system. It regulates the development, function, and differentiation of aberrant natural killer T cells as well as normal T cells. Ibrutinib inhibits T-cell differentiation–related ITK and BTK. Adding ibrutinib during the CAR-T cell creation process enhanced the proliferation of CAR-T cells and the durability of CAR-T cells derived from patients with chronic lymphocytic leukemia. Moreover, ibrutinib was used to select CAR-T cells that exhibited a less differentiated naïve-like phenotype and decreased expression of exhaustion markers such as PD-1, TIM-3, and LAG-3. Additionally, ibrutinib improved the ability of CAR cells generated from patients with chronic lymphocytic leukemia to secrete cytokines. To sum up, yield can be raised by ibrutinib-mediated BTK/ITK inhibition during CAR-T cell proliferation, as reported by.^63^

JAK2 is a nonreceptor tyrosine kinase that is involved in cell division, proliferation, and development, as well as histone changes, among other things. It regulates important signaling pathways that are involved in adaptive and innate immunity and plays a crucial role in signal transduction in the cytoplasm by binding to type I receptors (such as the growth hormone, prolactin, leptin, erythropoietin, thrombopoietin receptors), or type II receptors (such as receptors for IFN-α, IFN-β, IFN-γ, and many other interleukins).^64,65^ Herein, using T cell bispecific antibodies (TCB) and CARs targeting HER2, it was demonstrated that disrupting IFN-γ signaling provides resistance against death by activated T lymphocytes. Across multiple independently created resistant models, the kinase JAK2, which transduces the signal triggered by IFN-γ is frequently disturbed. Their findings reveal a fairly common tactic employed by cancer cells to thwart removal via misdirected lymphocytes.^66^

These receptors and kinases have various identified mutations and can impact CAR-T cell function; they can also be explored as targets to improve CAR-T cell into personalized cell therapy, reduce toxicity associated with CAR-T cell therapy, and increase efficacy.

## Conclusions and Perspectives

Proteomics is a great tool to understand protein mapping, protein–protein interactions, and the subcellular localization of the effectors of CAR-T cell therapy. Overall, we saw that CAR-T cell therapy utilizes the TCR signaling pathway and CAR-specific pathways, which are not yet fully characterized. With the continuous development of proteomics methods and technology, data reproducibility and precision in measurement with minimal noise will improve, and processes will become cheaper and faster. These enhancements will help shed more light on the critical players involved in molecular mechanism and signaling pathways of the cell therapy; it will also make room for unique protein signatures as prognostic biomarkers to measure treatment response and disease regression and allow the design of novel CAR constructs with great clinical benefits. Furthermore, it will become possible to identify the unique signature of a protein that could be targeted at the single-cell level to relieve the toxicity induced by the therapy, thereby enhancing the functionality of CAR-T cell therapy in nonsolid and solid tumors.

A limitation of this review is that proteomics methods in general exhibit limited sensitivity, resolution, reproducibility, and sample multiplexing, which are disadvantages for clinical applications.

We identified various molecular effectors and signaling pathways that play crucial roles in CAR-T cell function. The major ones include CD28, which promotes IL-2 production through the PI3K/Akt-dependent and -independent pathways; IL-5, which acts through the RAS-MAPK and JAK-STAT5 pathways; granzyme B, which mediates apoptosis by cleaving caspase-3, −9, and −10; LCK, which regulates the T-cell proliferative function via the TCR signaling pathways; and TNF-α, which induces cell death and IL-1 release, mediating the cytotoxic activity of the CAR-T cells. Receptors such as CD28, CD3E, and CD80 and the kinases B-Raf, ITK, and JAK2, among others, regulate the adaptive response of the immune system and regulate important signaling pathways. To sum up, the functions of these proteins include TCR activation, immune system activation and regulation, apoptosis signaling, and cytokine production regulation.

Despite these recent advances, CAR-T cell therapy remains the last resort for some patients. Usually, patients receiving this therapy have failed the first- or second-line of treatments. Therefore, the investment in even more personalized strategies to maximize the benefit of CAR-T cells is paramount, especially when considering the treatment cost. Further research is warranted on protein targets, such as the ones that we identified as key effectors, namely, CD28, IFNG, IL-2, IL-5, CCL3, granzyme B, LCK, TNF-α CD3E, CD80, BRAF, ITK, and JAK2. Such research can help improve the efficacy of the CAR-T cells and also identify patients who can benefit from cell therapy. We strongly believe that detailed molecular or protein effector profiles such as those discussed here will significantly improve CAR-T cell therapy. Some proteins, such as IFNG and IL-2, can be used as cytokine release syndrome markers, and others could be used as surrogate biomarkers^67^ to help predict patient response and manage side effects associated with CAR-T cell, including cytokine release syndrome and neurotoxicity.

## Supplementary Material



## Notes

Table S1: 79 proteins identified across proteomics studies with top 8 proteins highlighted and validated from at least 3 of the articles; Table S2: 438 modulated phosphoproteins that appeared at least twice out of the four data sets considered; Table S3: 45 proteins that are receptors and 71 kinases that play roles as molecular players of the CAR-T cell therapy from the phosphoproteomics studies (PDF)
