# Supplementary material for: A Proteomics Outlook on the Molecular Effectors of CAR‑T Cell Therapy in Cancer Management
Source: J Proteome Res. 2025 Mar 6;24(6):2571–83. doi: 10.1021/acs.jproteome.4c00930 (PMC12150318; doi:10.1021/acs.jproteome.4c00930)
Supplement: Supplementary file 1 [file pr4c00930_si_001.pdf]

A Proteomics Outlook on the Molecular Effectors of CAR-T Cell Therapy in Cancer Management

John Oluwafemi Teibo[1,2*], Virginia Picanço-Castro[2], Lucas Eduardo Botelho de Souza[2], Vitor Marcel Faça[1,2*]

[1]Department of Biochemistry and Immunology, Ribeirao Preto Medical School, University of São Paulo, Ribeirao Preto, São Paulo, 14049-900, Brazil. (johnteibo@usp.br; vitor.faca@fmrp.usp.br)

[2]Center for Cell-based Therapy CTC, Regional Blood Center of Ribeirão Preto, University of São Paulo, Ribeirão Preto, São Paulo, 14051-140, Brazil. (virginia.picanco@hemocentro.fmrp.usp.br; lucas.souza@hemocentro.fmrp.usp.br)

Corresponding Authors*: vitor.faca@fmrp.usp.br (Vitor Marcel Faça), johnteibo@usp.br (John Oluwafemi Teibo)

Supplementary Information

**STable 1**	**Proteomics Studies**	79 proteins identified across proteomics studies with top 8 proteins highlighted and validated from at least 3 of the articles
**STable 2**	**Phosphoproteomics studies**	438 modulated phosphoproteins that appeared at least twice out of the four datasets
**STable 3**	**Receptor and Kinases from Phosphoproteomics studies**	45 proteins that are receptors and 71 kinases that play roles as molecular effectors of the CAR-T cell therapy

**Supplementary Table 1**

79 proteins identified across proteomics studies with top 8 proteins highlighted and validated from at least 3 of the articles.

REGULATED PROTEINS FROM ALL 15 STUDIES	PROTEINS/GENE NAME	Number of Studies Observed
CCL5	CD28	5
GATA3	IFNG	5
HLA-B	IL2	4
IFNG	IL5	4
CCL3	CCL3	3
Cofilin-1	GZMB	3
IL10	LCK	3
LCK	TNF	3
CCL3	CCL4	2
CD28	CD96	2
GRB2	IL13	2
GZMB	IL17A	2
HLA-DRB3	IL32	2
HLA-F	IL4	2
IFNG	IL8	2
Abl1/2	Abl1/2	1
ACTG	ACTG	1
ACTIN1	ACTIN1	1
ACTN3	ACTN3	1
ACTR5	ACTR5	1
ADGRE2	ADGRE2	1
ANXA1	ANXA1	1
CCL3	CCL5	1
CCL4	CCR1	1
CCL4	CD-72	1
CCR1	CD3e	1
CD-72	CD40LG	1
CD28	CD6	1
CD28	CD70	1
CD28	CD8A	1
CD28	Cofilin-1	1
CD3e	ENO1	1

CD40LG	Erk1/2	1
CD6	FDXR	1
CD70	FLNA	1
CD8A	GATA3	1
CD96	GM-CSF	1
CD96	GRB2	1
ENO1	HLA-B	1
Erk1/2	HLA-DQB1	1
FDXR	HLA-DRB3	1
FLNA	HLA-F	1
GM-CSF	IL10	1
GZMB	IL16	1
GZMB	IL17A	1
HLA-DQB1	IL2RA	1
IFNG	IL9	1
IFNG	IL9	1
IFNG	LAT	1
IL13	LGALS3BP	1
IL13	LILRB2	1
IL16	MIP1α	2
IL17A	MIP1α	2
IL17A	MIP1β	1
IL17A	MSLN	1
IL2	MTCH1	1
IL2	NDUFS5	1
IL2	NFATC2	1
IL2	PCNA	1
IL2RA	Perforin	1
IL32	PKCq	1
IL32	PKM	1
IL4	PLCy1	1
IL4	Ribophorin-II	1
IL5	RRAS2	1
IL5	sCD137	1
IL5	SLP76	1
IL5	STAT3	1
IL8	Syntenin-1	1
IL8	TBX21	1
IL9	TCR	1

IL9	TGF-β	1
LAT	Th1 and Th2 cytokines	1
Lck	TMEM65	1
Lck	TNFRSF1B	1
LGALS3BP	TPI1	1
LILRB2	TRIP10	1
MIP1α	TRIP6	1
MIP1α	ZAP-70	1
MIP1β		
MSLN		
MTCH1		
NDUFS5		
NFATC2		
PCNA		
Perforin		
PKCq		
PKM		
PLCy1		
Ribophorin-II		
RRAS2		
sCD137		
SLP76		
STAT3		
Syntenin-1		
TBX21		
TCR		
TGF-β		
Th1 and Th2 cytokines		
TMEM65		
TNF		
TNF		
TNF		
TNFRSF1B		
TPI1		
TRIP10		
TRIP6		
ZAP-70		

Supplementary Table 2

438 modulated phosphoproteins that appeared at least twice out of the four datasets

Modulated Phosphoproteins	Number of Studies observed
CD3G	3
ADD3	3
BCL9L	3
BIN2	3
CRTC3	3
DKC1	3
DOT1L	3
EIF3C	3
EPB41	3
FGD3	3
KLC4	3
NCL	3
NIPBL	3
NOP2	3
NOP56	3
NUMA1	3
PDE3B	3
PITPNC1	3
RAB11FIP1	3
RAN	3
RIOK2	3
RPS6KA5	3
SAMSN1	3
SENP7	3
SIPA1	3
SMC4	3
SPEN	3
STK10	3
TBC1D4	3
WHAMM	3
ABCF1	2
ABLIM1	2
ACKR3	2

**ADA**	2
**AGFG1**	2
**ARAP1**	2
**ARFGAP2**	2
**ARHGAP35**	2
**CBLB**	2
**CCT4**	2
**CCT7**	2
**CD3D**	2
**CD3E**	2
**CD5**	2
**CFL1**	2
**CHERP**	2
**CTTN**	2
**DOCK2**	2
**DYRK3**	2
**ENO1**	2
**FASN**	2
**FKBP4**	2
**FLNB**	2
**GOLIM4**	2
**GPSM3**	2
**HSP90AA1**	2
**HSP90AB1**	2
**HSPA8**	2
**HSPD1**	2
**HSPE1**	2
**HSPH1**	2
**INPP5D**	2
**IPO5**	2
**IQSEC1**	2
**ITK**	2
**JAK2**	2
**LAT**	2
**LDHA**	2
**LMO7**	2
**LPXN**	2
**LRMP**	2
**LSP1**	2
**LYSMD2**	2

**MATR3**	2
**MCM2**	2
**MINK1**	2
**MYO1G**	2
**NEDD9**	2
**NUCKS1**	2
**PECAM1**	2
**PEX5**	2
**PGAM1**	2
**PKM**	2
**PLCG2**	2
**PLCL2**	2
**PTK2B**	2
**PXN**	2
**SERBP1**	2
**SHC1**	2
**SLAMF1**	2
**SLC12A4**	2
**SPRY1**	2
**SPTBN1**	2
**SWAP70**	2
**TCP1**	2
**THEMIS2**	2
**TNS3**	2
**TOP1**	2
**TPM2**	2
**TRANK1**	2
**TRIOBP**	2
**TUBB**	2
**TXNRD1**	2
**UNC119**	2
**VIM**	2
**AAK1**	2
**ACACA**	2
**AFAP1**	2
**AHCTF1**	2
**AKAP13**	2
**AKAP17A**	2
**AKAP2**	2
**AMPD3**	2

**ANAPC1**	2
**APBB2**	2
**ARHGAP12**	2
**ARHGAP17**	2
**ARID3A**	2
**ATP8A1**	2
**BAD**	2
**BCCIP**	2
**BIN1**	2
**BIVM-ERCC5**	2
**BOD1L1**	2
**BOP1**	2
**BRAF**	2
**BRD1**	2
**BYSL**	2
**BZW2**	2
**CAD**	2
**CAMK4**	2
**CAT**	2
**CBFB**	2
**CBX7**	2
**CCDC14**	2
**CCDC43**	2
**CCDC86**	2
**CD3EAP**	2
**CD80**	2
**CDC25B**	2
**CDC26**	2
**CDK9**	2
**CEBPB**	2
**CEP170B**	2
**CEP55**	2
**CHD1**	2
**CLN6**	2
**CTNNA1**	2
**CTPS1**	2
**CTPS2**	2
**CTU2**	2
**DAXX**	2
**DCK**	2

**DDX21**	2
**DEK**	2
**DENND4C**	2
**DGCR14**	2
**DNAJC2**	2
**DNM2**	2
**DOCK10**	2
**DOK4**	2
**DUS3L**	2
**DYNC1LI2**	2
**EDC4**	2
**EEPD1**	2
**EIF3A**	2
**EIF3B**	2
**EIF3K**	2
**EIF4B**	2
**EIF4G1**	2
**EIF5B**	2
**EIF6**	2
**ELAVL1**	2
**EOMES**	2
**ESF1**	2
**EVL**	2
**EXOC4**	2
**FAF1**	2
**FAM117A**	2
**FAM122A**	2
**FAM129B**	2
**FAM214A**	2
**FARP2**	2
**FBXL20**	2
**FTSJ3**	2
**FUBP3**	2
**GEMIN5**	2
**GFPT2**	2
**GLCCI1**	2
**GMIP**	2
**GNL2**	2
**GRAMD4**	2
**GRWD1**	2

**GTPBP4**	2
**HERC1**	2
**HIST1H1C**	2
**HIVEP3**	2
**HLA-A**	2
**HMGA1**	2
**HNRNPA2B1**	2
**HNRNPA3**	2
**HRH1**	2
**IKZF1**	2
**ILF3**	2
**IMPDH2**	2
**INCENP**	2
**INTS1**	2
**IWS1**	2
**KCTD15**	2
**KDM3B**	2
**KIAA0319L**	2
**KIF23**	2
**KIF4A**	2
**KLC1**	2
**KLF12**	2
**KLF2**	2
**KMT2D**	2
**LARP1B**	2
**LARP4**	2
**LEF1**	2
**LEMD2**	2
**LEO1**	2
**LLGL1**	2
**LRRFIP1**	2
**LTV1**	2
**MAK16**	2
**MAP1A**	2
**MAP7D1**	2
**MBD5**	2
**MIS18BP1**	2
**MKI67**	2
**MLF2**	2
**MLLT11**	2

**MYBBP1A**	2
**MYC**	2
**MYO18A**	2
**MYO1F**	2
**NAA15**	2
**NACA**	2
**NACC1**	2
**NAF1**	2
**NCF2**	2
**NCOA7**	2
**NCOR1**	2
**NDRG3**	2
**NEK6**	2
**NELFB**	2
**NELFE**	2
**NIPAL3**	2
**NOC2L**	2
**NOLC1**	2
**NONO**	2
**NOP14**	2
**NOP58**	2
**NPM1**	2
**NSUN2**	2
**NUFIP1**	2
**OSBPL11**	2
**OSBPL3**	2
**P2RY8**	2
**PA2G4**	2
**PACS1**	2
**PAICS**	2
**PANK4**	2
**PASK**	2
**PBXIP1**	2
**PCF11**	2
**PCMTD1**	2
**PCNT**	2
**PDCD4**	2
**PDE7A**	2
**PDIA6**	2
**PES1**	2

**PFAS**	2
**PFKP**	2
**PHF14**	2
**PHF21A**	2
**PHF3**	2
**PHKB**	2
**PIAS1**	2
**PLCB3**	2
**PLEKHA1**	2
**PNISR**	2
**POGZ**	2
**POP1**	2
**PPFIA1**	2
**PRKAB2**	2
**PRKCB**	2
**PRKCH**	2
**PRKD3**	2
**PRRC2C**	2
**PRSS23**	2
**PSIP1**	2
**PTPN7**	2
**PUM2**	2
**PUS1**	2
**QRICH1**	2
**RABEP1**	2
**RANBP1**	2
**RANGAP1**	2
**RAPGEF1**	2
**RASGRP2**	2
**RBBP6**	2
**RBL2**	2
**RBM14**	2
**RBM25**	2
**RBM39**	2
**RCAN1**	2
**RCSD1**	2
**RFX3**	2
**RIOK1**	2
**RMDN2**	2
**RNF213**	2

**RPL22L1**	2
**RPS6KA1**	2
**RRAS2**	2
**RRP12**	2
**RRP15**	2
**RRP9**	2
**RUNX1**	2
**SAFB**	2
**SAP30L**	2
**SCRIB**	2
**SEC22B**	2
**SEC61B**	2
**SELPLG**	2
**SGTB**	2
**SH2D3C**	2
**SIDT2**	2
**SIGIRR**	2
**SIK3**	2
**SIPA1L1**	2
**SLC16A1**	2
**SLC35F2**	2
**SLC38A1**	2
**SLC4A4**	2
**SLC4A7**	2
**SLK**	2
**SMARCAD1**	2
**SMARCC2**	2
**SNTB2**	2
**SON**	2
**SPATA5L1**	2
**SPIDR**	2
**SRFBP1**	2
**SRRM1**	2
**SRRM2**	2
**SRSF10**	2
**SRSF11**	2
**SSBP2**	2
**STIP1**	2
**STK38**	2
**STMN1**	2

**STRAP**	2
**SYNJ2**	2
**TBC1D1**	2
**TBC1D10C**	2
**TBC1D22A**	2
**TELO2**	2
**TFDP2**	2
**TFPT**	2
**TIAM2**	2
**TLE3**	2
**TMA16**	2
**TMEM87A**	2
**TMX4**	2
**TNRC6C**	2
**TOM1L2**	2
**TOP2B**	2
**TP53BP1**	2
**TRAFD1**	2
**TRIM24**	2
**TTLL12**	2
**TXNIP**	2
**U2SURP**	2
**UCHL3**	2
**UCK2**	2
**UHRF1BP1L**	2
**UIMC1**	2
**UTP14A**	2
**UTP18**	2
**UTP20**	2
**UTRN**	2
**WARS**	2
**WDR3**	2
**WDR43**	2
**WDR62**	2
**WDR7**	2
**WEE1**	2
**YRDC**	2
**YWHAZ**	2
**ZC3H11A**	2
**ZC3HAV1**	2

**ZFAND2B**	2
**ZFP36L2**	2
**ZMYM3**	2
**ZNF609**	2
**ZNRF1**	2
**ZRANB2**	2
**ABCC5**	2
**AHNAK**	2
**ALG3**	2
**ARL11**	2
**ATAD3B**	2
**BCL2L1**	2
**BTN3A1**	2
**BTN3A2**	2
**BTN3A3**	2
**C16orf54**	2
**CEP162**	2
**FAM102A**	2
**FYB**	2
**GTF2IRD2**	2
**GTF2IRD2B**	2
**GZMB**	2
**HEATR3**	2
**IL4R**	2
**KIAA0430**	2
**KIAA1671**	2
**KRT81**	2
**LRCH4**	2
**MTFP1**	2
**NHP2**	2
**NT5DC1**	2
**PHC1**	2
**PPP1R12B**	2
**SLC7A5**	2
**SORL1**	2
**SSH3**	2
**SYCP2**	2
**TXN**	2
**UBASH3B**	2
**CD247**	2

**CD28**	2
**HLA-C**	2
**MGA**	2
**RAPGEF2**	2
**STK17B**	2

Supplementary Table 3

45 proteins that are receptors and 71 kinases that play roles as molecular effectors of the CAR-T cell therapy

Receptor FINAL LIST	Kinase FINAL LIST
CD3G	ADD3
NCL	PDE3B
ACKR3	RIOK2
ARAP1	RPS6KA5
CBLB	STK10
CD3D	CBLB
CD3E	CD3E
CD5	DYRK3
DOCK2	HSP90AA1
FKBP4	HSP90AB1
HSP90AA1	ITK
HSP90AB1	JAK2
HSPA8	LAT
IPO5	MINK1
LAT	PGAM1
PECAM1	PKM
PLCL2	PLCG2
SHC1	SHC1
SLAMF1	SLC12A4
CD80	TOP1
DAXX	AAK1
EIF3A	AKAP13
HLA-A	AKAP17A
HRH1	BCCIP
ILF3	BRAF
LEF1	CAD
NCOA7	CAMK4
NCOR1	CDC25B
P2RY8	CDK9
RBM14	DCK
SEC22B	EIF3A
SELPLG	ELAVL1
SIPA1L1	FAF1
STRAP	LLGL1
TRIM24	NAF1
BTN3A1	NEK6

BTN3A2	NPM1
BTN3A3	PANK4
FYB	PASK
IL4R	PFKP
SORL1	PHKB
CD28	PRKAB2
HLA-C	PRKCB
RAPGEF2	PRKCH
CD247	PRKD3
	RBBP6
	RIOK1
	RPS6KA1
	SCRIB
	SIK3
	SLK
	STK38
	TELO2
	TFPT
	TOM1L2
	TRIM24
	UCK2
	UTRN
	WARS
	WEE1
	YWHAZ
	BCL2L1
	PPP1R12B
	CD247
	CD28
	STK17B
	NEDD9
	PTK2B
	BAD
	CEBPB
	DAXX
